# N-mixture model-based estimate of relative abundance of sloth bear (*Melursus ursinus*) in response to biotic and abiotic factors in a human-dominated landscape of central India

**DOI:** 10.7717/peerj.13649

**Published:** 2022-12-06

**Authors:** Sankarshan Chaudhuri, Rajasekar Rajaraman, Sankar Kalyanasundaram, Sambandam Sathyakumar, Ramesh Krishnamurthy

**Affiliations:** 1 Department of Landscape Level Planning and Management, Wildlife Institute of India, Dehradun, Uttarakhand, India; 2 Salim Ali Centre for Ornithology and Natural History, Coimbatore, Tamil Nadu, India; 3 Department of Endangered Species Management, Wildlife Institute of India, Dehradun, Uttarakhand, India; 4 Faculty of Forestry, University of British Columbia, Vancouver, British Columbia, Canada

**Keywords:** Sloth bear, Relative abundance, Camera trap, N-mixture model, Human-dominated landscape

## Abstract

Reliable estimation of abundance is a prerequisite for a species’ conservation planning in human-dominated landscapes, especially if the species is elusive and involved in conflicts. As a means of population estimation, the importance of camera traps has been recognized globally, although estimating the abundance of unmarked, cryptic species has always been a challenge to conservation biologists. This study explores the use of the N-mixture model with three probability distributions, *i.e*., Poisson, negative binomial (NB) and zero-inflated Poisson (ZIP), to estimate the relative abundance of sloth bears (*Melursus ursinus*) based on a camera trapping exercise in Sanjay Tiger Reserve, Madhya Pradesh from December 2016 to April 2017. We used environmental and anthropogenic covariates to model the variation in the abundance of sloth bears. We also compared null model estimates (mean site abundance) obtained from the N-mixture model to those of the Royle-Nichols abundance-induced heterogeneity model (RN model) to assess the application of similar site-structured models. Models with Poisson distributions produced ecologically realistic and more precise estimates of mean site abundance (*λ* = 2.60 ± 0.64) compared with other distributions, despite the relatively high Akaike Information Criterion value. Area of mixed and sal forest, the photographic capture rate of humans and distance to the nearest village predicted a higher relative abundance of sloth bears. Mean site abundance estimates of sloth bears obtained from the N-mixture model (Poisson distribution) and the RN model were comparable, indicating the overall utility of these models in this field. However, density estimates of sloth bears based on spatially explicit methods are essential for evaluating the efficacy of the relatively more cost-effective N-mixture model. Compared to commonly used index/encounter-based methods, the N-mixture model equipped with knowledge on governing biotic and abiotic factors provides better relative abundance estimates for a species like the sloth bear. In the absence of absolute abundance estimates, the present study could be insightful for the long-term conservation and management of sloth bears.

## Introduction

Knowledge of the abundance of a species is fundamental for ecological studies and a prerequisite for successful conservation planning and decision-making processes in the field of conservation biology (Kremen, Merenlender & Murphy, 1994; Gaston & Rodrigues, 2003; Nichols & Williams, 2006; McCarthy & Possingham, 2007). Reliable abundance estimates can also be crucial for identifying the biotic and abiotic factors that govern the population size (Meents et al., 1983). Inaccurate estimates or estimates based on empirical studies can lead to erroneous management decisions and undermine the effort to conserve the concerned species in its natural environment. However, estimating the abundance of cryptic species through the traditional capture-mark-recapture (CMR) method is labor-intensive and is not always logistically feasible. Exploration and subsequent implementation of cost-effective methodologies to monitor population abundance are thus required, given the limited resource availability in the field of wildlife conservation (Parker et al., 2011).

With the advent of science and technology, passive detectors, especially automated camera traps, have become indispensable in wildlife and conservation biology (O’Connell, Nichols & Karanth, 2011; Burton et al., 2015). However, despite the diverse applications of camera traps, their use has been confined to population estimation of individually identifiable or marked animals (Burton et al., 2015). Camera trap surveys provided successful population density estimates through spatial capture-recapture (SCR) models for marked animals (Karanth & Nichols, 1998; O’Connell, Nichols & Karanth, 2011). However, population estimation for unmarked or individually unidentifiable animals *via* camera trapping has posed challenges to ecologists and conservation biologists (Gilbert et al., 2021). Also, for unmarked species like Asian bears, population monitoring in a small area (<10,000 km^2^) requires spatially explicit methods (*e.g*., camera traps or genetics), but these studies remain restricted due to resource and logistical constraints (Proctor et al., 2022).

Photographic capture rates (number of individuals photo-captured/effort in terms of trap-nights) or relative abundance indices provide surrogates of population abundance (Carbone et al., 2001; O’Brien, Kinnaird & Wibisono, 2003; Bengsen et al., 2011). However, inferences drawn from these index-based surveys do not meet the implicit assumption of equal detection probability (Jennelle, Runge & MacKenzie, 2002; Harmsen et al., 2010; Sollmann et al., 2013; Burton et al., 2015). Rowcliffe et al. (2008) proposed the random encounter model (REM) to estimate density by incorporating information on animal movement, photographic capture rate and the detection zone of camera traps. Despite its unique and robust approach, the limited application of REM is due to the actual random deployment of camera traps and the inability to account for spatial variation of abundance. Also, the need to extrapolate density from the camera trap detection zone to the entire surveyed area, and the lack of knowledge regarding the movement of elusive species, further restricts its applicability (Gilbert et al., 2021). The application of SCR frameworks for unmarked individuals, otherwise known as unmarked spatial capture-recapture (USCR; Chandler & Andrew Royle, 2013), is another promising approach to estimating population density. However, its limited applicability is due to the underlying computationally intensive (Bayesian) framework (Royle et al., 2014; Gilbert et al., 2021), tendency to produce imprecise estimates (Augustine et al., 2019) and judicious selection of priors on the spatial scale of animal detection at camera traps (Sun, Fuller & Royle, 2014). On the other hand, site-structured models such as the Royle-Nichols abundance-induced heterogeneity model (RN model; Royle & Nichols, 2003) and the N-mixture model (Royle, 2004) have been widely used to draw inferences on abundance from detection-nondetection data and count data, respectively, based on spatially or temporally replicated surveys. In the recent past, the latter was more widely applied to estimate the abundance of terrestrial mammals from count data (Keever et al., 2017; Xiao et al., 2018; Kafley et al., 2019; Kidwai et al., 2019; Penjor et al., 2019; Searle et al., 2020). The major advantages of the N-mixture model are its cost-effectiveness, the ability to bypass individual identification, and the ability to account for spatial variation of abundance as a function of covariates. The N-mixture model typically requires the following assumptions: population closure at the site, equal probability of detection of all individuals at a site, no false-positive errors (*i.e*., misidentification or double-counting of individuals), and independent detection of individuals across the sampling units (Royle, 2004). However, N-mixture models are sensitive to assumption violations, especially false-positive errors, which are not commonly addressed (Kéry & Schaub, 2011). For free-ranging unmarked species, one individual would likely be photo-captured several times during the entire sampling occasion (O’Brien, Kinnaird & Wibisono, 2003), which can overestimate abundance (Link et al., 2018; Nakashima, 2020). However, relaxing the assumption of population closure at the site for free-ranging species, it is justifiable to interpret the true abundance of the site as “relative abundance,” which translates to the number of individuals utilizing a site at a given time (Kéry & Royle, 2015).

The sloth bear (*Melursus ursinus*), one of the four bear species found in the Indian subcontinent, is mainly confined to the isolated patches of forested habitat both inside and outside of the protected areas (PAs) (Sathyakumar et al., 2012; Dharaiya, Bargali & Sharp, 2016; Jhala, Qureshi & Nayak, 2020). Despite the species’ broad geographic distribution, especially across the moist and dry deciduous forests of the Western Ghats and central Indian landscape (Yoganand et al., 2006), information on population abundance based on a rigorous statistical framework is lacking (Dharaiya, Bargali & Sharp, 2016). However, the range-wide population abundance of sloth bears reportedly ranges between 10,000 and 20,000 (Garshelis, Joshi & Smith, 1999; Chauhan, 2006; Yoganand et al., 2006; Sathyakumar et al., 2012). None of these estimates was reliable enough to monitor the population trends because of the differential data collection methods and types of data used in the analyses (Sathyakumar et al., 2012; Dharaiya, Bargali & Sharp, 2016). The sloth bear is an omnivorous generalist, long-ranging ursid and often comes in conflict with humans (Rajpurohit & Krausman, 2000; Bargali, Akhtar & Chauhan, 2005; Garcia, Joshi & Dharaiya, 2016; Debata et al., 2017; Dhamorikar et al., 2017; Singh, Sonone & Dharaiya, 2018). The ever-increasing human population, subsequent habitat fragmentation and human-sloth bear conflicts are significant concerns for conserving this species. One recent study (Gomez et al., 2021) also indicated a steadily increasing trend of sloth bear mortality due to poaching. So far, only one study (Garshelis, Joshi & Smith, 1999) has attempted to make robust density estimates of the sloth bear, done in Royal Chitwan National Park (henceforth Chitwan), Nepal, based on a spatial mark-resight method. Reliable information on population abundance and identifying factors influencing abundance are crucial for the long-term conservation of sloth bears in human-dominated landscapes.

In this context, we explored the application of site-structured models with special reference to the N-mixture model (Royle, 2004) to quantify the relative abundance of sloth bears in Sanjay Tiger Reserve, Madhya Pradesh. The objectives of the present study were as follows: (1) to demonstrate the use of the N-mixture model in estimating the relative abundance of sloth bears and compare the mean site abundance estimates obtained from the N-mixture model to those of the RN model and (2) to identify the environmental and anthropogenic covariates that govern the relative abundance of sloth bears.

## Materials and Methods

### Research permissions and ethical considerations

Madhya Pradesh Forest Department (MPFD) issued all required permissions for our field surveys (Letter No: I/3129 dated 29/05/2014). Due to the non-invasive nature of sampling, we did not require ethical clearance for this study.

### Study site

**Figure 1:**
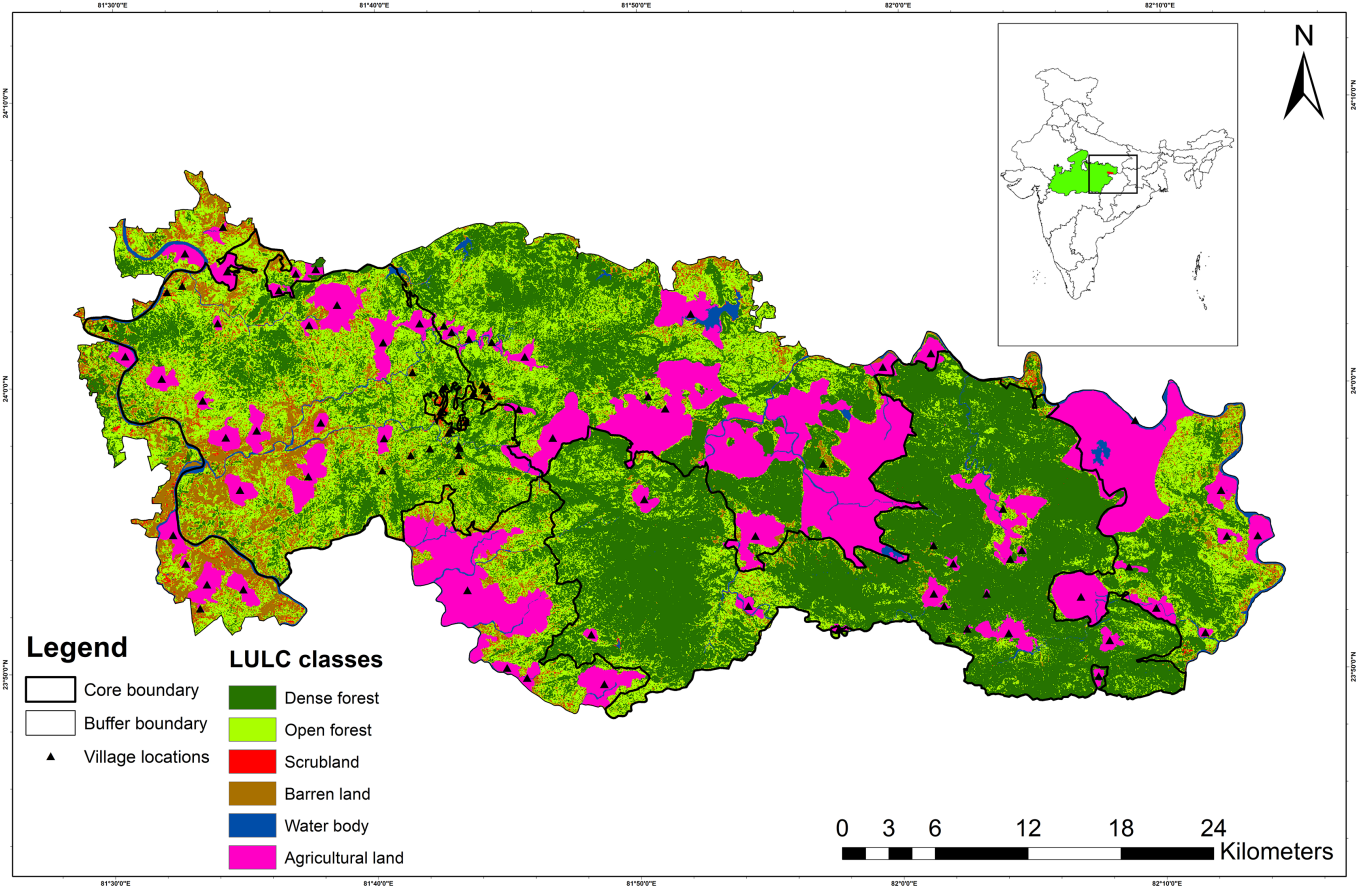
Map showing Sanjay Tiger Reserve with land use land cover (LULC) classes and location of villages; Inset shows the location of Sanjay Tiger Reserve in Madhya Pradesh, India.

Sanjay Tiger Reserve (STR) is situated between 24.125122°N 81.546009°E (northwestern boundary) and 23.822641°N 82.175139°E (southeastern boundary) in the state of Madhya Pradesh in central India (Fig. 1). STR falls under the category “Central Highlands” as per the biogeographic classification of India (Rodgers & Panwar, 1988). The core and buffer areas of STR encompass 831.25 km^2^ and 812.58 km^2^, respectively. Furthermore, the core area of STR consists of two administrative parts, *i.e*., Sanjay National Park (SNP; 466.60 km^2^) and Dubri Wildlife Sanctuary (DWLS; 364.60 km^2^).

The terrain of STR is rugged and undulating, with plateaus and gorges in the eastern part (SNP) and flat land in the western part (DWLS). STR has an elevation range between 425 and 732 m. The broad forest type of STR falls under sub-group 3C- “North Indian moist deciduous forests”, with subtype (C2) “Moist sal-bearing forest” with subdivision (2e) “Moist peninsular sal forest” (Champion & Seth, 1968). Sal (*Shorea robusta*) is dominant in SNP and DWLS, occupying about 80% of the entire area, followed by mixed, bamboo mixed, scrubland and grassland (Fig. S1). Apart from sal, other frequently found tree species include tendu (*Diospyros melanoxylon*), mahua (*Madhuca longifolia*), char (*Buchanania cochinchinensis*) and sedha (*Lagerstroemia parviflora*). STR receives a mean annual precipitation of 1,303 mm between June and September, and the annual temperature varies between 7.4 °C and 41.8 °C. Other than the sloth bear, tiger (*Panthera tigris*), leopard (*Panthera pardus*), striped hyena (*Hyaena hyaena*), Indian wolf (*Canis lupus*) and Asiatic wild dog (*Cuon alpinus*) are the major carnivores found in STR. Anthropogenic pressure is immense inside the core area of STR due to the presence of thirty-nine villages.

### Camera trap survey

The camera trapping survey was conducted from December 2016 to April 2017 in the core area of STR. We divided the study area into 2 km × 2 km grids (henceforth, sites) following the All India Tiger Estimation protocol (Jhala, Qureshi & Gopal, 2015) and subsequently deployed camera traps in 143 locations (one at each site). We divided the core area into two blocks (SNP and DWLS), and camera traps were deployed in one block at a time due to the limited availability of camera traps. A pair of motion-triggered camera traps (Cuddeback C1 and Cuddeback Ambush) were deployed at each site opposite to one another to increase the detection (Pease, Nielsen & Holzmueller, 2016; O’Connor et al., 2017) at an approximate height of 30–40 cm above the ground. We placed the camera traps on forest roads, animal trails and dry riverbeds to maximize the photo-capture of sloth bears and other large carnivores (Karanth et al., 2011). The average distance between two adjacent camera trap locations was 2 km. We set 15 s time-lapse between two consecutive photographs. Cameras were active 24 h a day for an average trapping period of 42 days (range: 13–45 days) and checked once every 20–25 days to change batteries and memory cards. We collected the locations of camera traps by using Garmin e-Trex handheld GPS units (Garmin Inc., Olathe, KS, USA).

To avoid pseudoreplication, we considered two consecutive photographs of sloth bears or humans captured ≥30 min apart as independent events (O’Brien, Kinnaird & Wibisono, 2003; Xiao et al., 2018; Kafley et al., 2019). We set the temporal sampling unit (sampling occasion) as three days. We pooled the count of independent photographs of sloth bears from each site for each unit of three consecutive days, which yielded an encounter history of 15 sampling occasions. Reducing the sampling period by pooling data from a specific time (days) helped reduce overdispersion (Penjor et al., 2019).

### Covariates

#### Local-scale spatial covariates

At each camera trap location, we measured habitat features within a 10 m-radius plot, with a focus on fruiting trees (≥2 m in height and ≥1 m girth at breast height), which comprise ≥1% of the diet of sloth bears (Ramesh et al., 2012; Rather et al., 2020). We also recorded the number of termite mounds and woody shrub species at every 10 and 5 m-radius plot, respectively. We calculated the photo-capture rate of humans (number of photographs of humans at each location/number of days each camera trap was active) as one of the surrogates of anthropogenic pressure at each site. Table 1 provides the details of local-scale covariates.

**Table 1:** List of covariates to model the relative abundance of sloth bears using the N-mixture model in Sanjay Tiger Reserve, Madhya Pradesh, India, during 2016–2017.

A. Environmental covariates (with abbreviations)	Description	Range of values	Area
Land use land cover (LULC) classes	LULC was classified into six classes: dense forest, open forest, scrubland, barren land, agricultural land and water body. Area (in km^2^) of each class was calculated and extracted for each 12.40 km^2^ grid in Arc GIS 10.2.	1–6 (Number of classes)	Dense forest-706.66 km^2^ Open forest-447.20 km^2^ Scrubland-41.81 km^2^ Barren land-66.77 km^2^ Agricultural land-303.25 km^2^ Water body-37.95 km^2^
Forest type classes	Forest type map was classified into six classes: sal mixed, mixed, sal forest, bamboo mixed, grassland and non-forest. Area (in km^2^) of each class was calculated and extracted for each 12.40 km^2^ grid in Arc GIS 10.2.	1–6 (Number of classes**)**	Sal mixed-538.06 km^2^ Mixed-457.54 km^2^ Sal forest-150.03 km^2^ Bamboo mixed-55.65 km^2^ Grassland-7.28 km^2^ Non-forest-390.02 km^2^
Distance to water source (km)	Euclidean distance to nearest water source (natural or artificial) from the centroid of each 12.40 km^2^ grid was calculated using the Spatial analyst tool in Arc GIS 10.2.	0–15	**-**
Ruggedness index	Ruggedness index was generated for each 12.40 km^2^ grid, from DEM (Digital Elevation Model) by the roughness index tool in the 3D Analyst extension in Arc GIS 10.2.	9.21–27.61	**-**
Availability (or density) of fruiting trees (no./m^2^)	Number of fruiting trees was counted per 10 m-radius plot around each camera trap location and subsequently divided by the area (in m^2^) of each plot.	0–0.09	**-**
Density of shrubs (no./m^2^)	Number of woody shrubs was counted per 5 m-radius plot around each camera trap location and subsequently divided by the area (in m^2^) of each plot.	0–1.39	
No. of termite mounds	Number of termite mounds was counted per 10 m-radius plot around each camera trap location.	0–4	**-**
**B. Anthropogenic covariates (with abbreviations)**			
Human photo-capture rate (no./total effort)	Number of total photographs of humans obtained from each camera trap was counted and divided by the total effort (trap-days or trap-nights) of each camera trap	0–11.87	**-**
Distance to village (km)	Euclidean distance to nearest village from the centroid of each 12.40 km^2^ grid was calculated using the Spatial analyst tool in Arc GIS 10.2.	0–12	**-**
Distance to metal road (km)	Euclidean distance to nearest metal road from the centroid of each 12.40 km^2^ grid was calculated using the Spatial analyst tool in Arc GIS 10.2.	0–14	**-**

#### GIS-based covariates

We prepared the Land Use Land Cover (LULC) layer for STR by processing a 30 m spatial resolution Landsat-8 image (Image ID: LC81430432016297LGN00) dated 23 October 2016 in ERDAS IMAGINE 9.2 (Leica Geosystems, St. Gallen, Switzerland) following a standard hybrid image classification protocol (Ruppert et al., 1997). The image classification resulted in six classes, *i.e*., dense forest, open forest, scrubland, agricultural land, barren land, and water bodies. The average accuracy of the resultant classified image was 88.89%, and the overall Kappa coefficient was 0.85. To classify the forest types in STR, we processed a 30 m spatial resolution Landsat-8 image (Image ID: LC81430432018110LGN00) dated 2 May 2018, following the methodology mentioned above and with the support of 412 ground points. The classes included sal mixed, mixed, sal forest, bamboo mixed, grassland and non-forest. We categorized the different forest types following the broad classification by Champion & Seth (1968). Subsequently, we performed the accuracy assessment of the resultant classified image based on 392 ground points collected throughout the study area. This classified image’s average accuracy and overall Kappa coefficient were 64.30% and 0.45, respectively.

Other covariates used in the analyses were the terrain ruggedness index (RI; Riley, 1999) and the distances to the nearest water source, metal road and village (see Table 1 for details). All analyses were carried out in ERDAS IMAGINE 9.2 (Leica Geosystems, St. Gallen, Switzerland) and ArcGIS 10.2 (Environmental Systems Research Institute, Redlands, CA, USA).

### Relative abundance model of sloth bear

We developed the N-mixture model (Royle, 2004; Kéry & Royle, 2015) to estimate the relative abundance of sloth bears in STR within 3.52 km × 3.52 km grids, as this represents the minimum home range (*i.e*., 12.40 km^2^) of sloth bear in central India (Yoganand, 2005). However, little is known about the home range size of the sloth bear in a human-dominated landscape, though studies elsewhere (Joshi, Garshelis & Smith, 1995; Ratnayeke, Manen & Padmalal, 2007) have indicated much smaller average home range estimates compared to those of the central Indian landscape (Yoganand, 2005). Since we deployed camera traps within smaller grids (2 km × 2 km), we used the data from all 1–4 camera traps falling inside each 3.52 km × 3.52 km grid and considered these as spatial replicates, following Xiao et al. (2018). We used the N-mixture model to estimate the relative abundance of sloth bears from independent photographic captures as a function of environmental and anthropogenic covariates (Table 1).

N-mixture models assume population closure (*i.e*., the number of individuals residing within a site is constant as no emigration or immigration occurs between sites). Due to the individually unidentifiable aspect and long-ranging behavior of the sloth bear, we believe that the closure assumption was likely to be violated. Hence, we relaxed the closure assumption by changing the interpretation from absolute abundance at a site to the number of individuals ever associated with a site during a given period (Kéry & Royle, 2015), otherwise known as relative abundance. We pooled independent detections of sloth bear for each site “i” from “R” sites where, i = 1, 2, 3,..., R during a sampling occasion of “T”, where t = 1, 2, 3,…, T. The observed count (y_it_) at “i” sites during “t” sampling occasions followed a binomial distribution, and the site abundance N_i_ followed a Poisson distribution (Kéry & Royle, 2015).

Due to the greater flexibility in modeling abundance with covariates and the ability to incorporate other effects (*e.g*., zero inflation, latent state), Poisson distribution is an integral part of the state process, *i.e*., abundance (Royle & Dorazio, 2008). On the other hand, binomial distribution accounts for imperfect detection or false-negative errors. The variation of observed counts (y_it_) is an effect of imperfect detection of the actual (unknown) abundance N_i_ and its variability among sites. In simple algebraic form, we expressed the two processes as:

State process: N_i_ ~ Poisson (*λ*_i_); log (*λ*_i_) = β_0_ + effects of covariates

Observation process: y_it_ | N_i_ ~ Binomial (N_i_, p_it_); logit (p_it_) = α_0_ + effects of covariates

where N_i_ is the latent abundance of site “i” (i = 1,…., R), *λ*_i_ is the mean abundance of site “i”, y_it_ is the count of the species at site “i” and occasion “t” (t = 1,…., T), and p_it_ is detection probability at site “i” and occasion “t”.

Studies have indicated that N-mixture models with Poisson and zero-inflated Poisson (ZIP) distributions can produce reliable relative abundance estimates if detection probability is modelled effectively with appropriate covariates (Barker et al., 2018; Kéry, 2018). We modelled the detection probability with the trapping period as we suspected that a more extended trapping period would positively influence the detection probability of sloth bears (O’Connor et al., 2017). We also used local-scale spatial covariates (and no GIS-based covariates) likely to affect the detection probability, following Hofmeester et al. (2019). The detection probability would be constant if none of the covariates influenced it (Kafley et al., 2019).

After determining the effect of covariates on the detection process, we modelled the relative abundance of sloth bears as a function of environmental (forest types, LULC types, RI, distance to nearest water source, availability of fruiting trees, shrub density and number of termite mounds) and anthropogenic (human photographic capture rate, distance to nearest village and metal road) covariates with three probability distributions, *i.e*., Poisson, ZIP and negative binomial (NB). Mean values of GIS-based environmental and anthropogenic covariates were calculated and extracted from each of the 12.40 km^2^ grids. For local-scale spatial covariates where the number of camera trap locations was more than one, we calculated the average values of each covariate for the 12.40 km^2^ grids. The selection of the most parsimonious models from the candidate model set followed the ascending Akaike Information Criterion (AIC) values (Burnham & Anderson, 2002). We reported uninformative parameters but drew final inferences only from the models within ≤2 ΔAIC units (Burnham & Anderson, 2002) with statistically significant (*P* < 0.05) parameters (Arnold, 2010). Model averaging was not considered in the case of a single top-ranked model or the presence of uninformative parameters in multiple top-ranked models (Arnold, 2010). However, due to the “good fit bad prediction dilemma” as mentioned by Kéry & Royle (2015), we further performed Goodness of Fit (GoF) tests with 1,000 iterations of bootstrapping for each of the most parsimonious models of corresponding distributions. We also calculated overdispersion parameters (ĉ) for the same models (Kéry & Royle, 2015). We proceeded with further residual diagnostics and mapping of residuals if none of them passed the GoF test (when *P* < 0.05) for the final checking of model adequacy (Kéry & Royle, 2015). The result was interpreted as an outcome of unstructured noise if no specific pattern of lack of fit was found (Kéry & Royle, 2015). Subsequently, we corrected it by inflating the predicted abundance’s range (confidence interval) (Johnson, Laake & Ver Hoef, 2010; Kéry & Royle, 2015). We standardized all covariates using Z-transformation before analyses, and subsequently, Pearson’s correlation tests were performed to avoid multi-collinearity. A pairwise Pearson’s correlation coefficient value of r≥ ± 0.7 was considered strongly correlated (Dormann et al., 2013) and was not modeled together, as we were interested in seeing the effect of each covariate on the relative abundance of sloth bears. All analyses were carried out using the packages “Unmarked” (Fiske & Chandler, 2011) and “AICmodavg” (Mazerolle, 2015) implemented in R (R Core Team, 2019).

We compared the mean site abundance estimates with estimates obtained from another frequently used site-structured model, the RN model (Royle & Nichols, 2003), to understand the performance of the N-mixture model. Since modeling the relative abundance of the sloth bear with both local-scale and GIS-based covariates by applying the RN model and subsequent comparison of the model performance (of both N-mixture and RN models) was beyond the scope of this paper, we restricted the comparison between the null models of the two frameworks mentioned above. We chose the same probability distribution for the RN model which was the most suitable for the N-mixture model. We used the program Presence (Version 2.13.11) (Hines, 2006) to estimate the mean site abundance of sloth bears by applying the RN model.

## Results

### Camera trap

**Figure 2:**
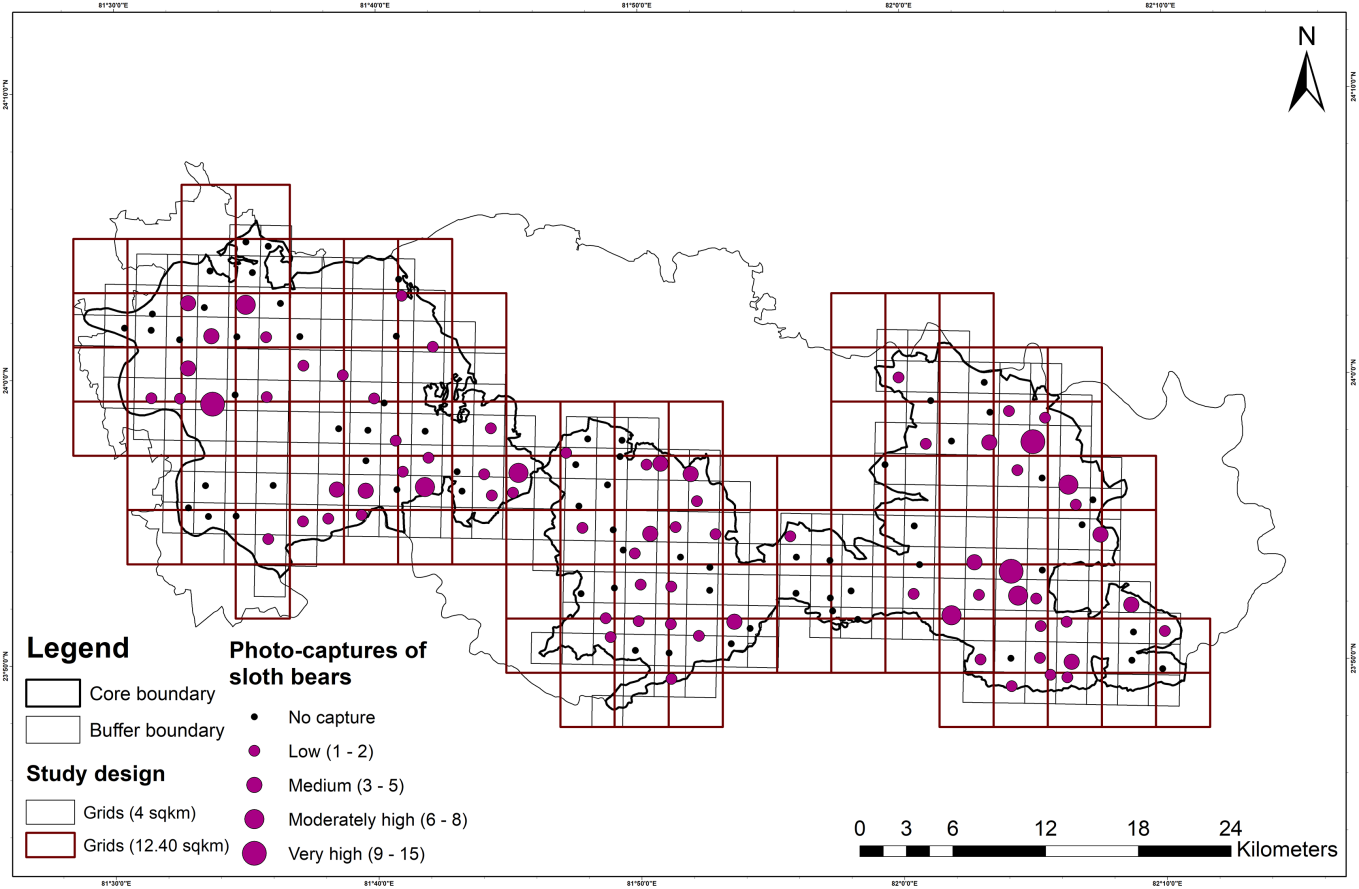
Map showing the study design, followed in Sanjay Tiger Reserve, Madhya Pradesh, India. It includes the larger sampling units, *i.e*., 12.40 km^2^ grids, fine-scale sampling units, *i.e*. 4 km^2^ grids and number of photo-captures of sloth bears.

A total of 191 and 5,292 independent photographs of sloth bears and humans, respectively, were obtained from a total effort of 5,950 trap-nights (3,870 and 2,080 trap-nights in SNP and DWLS, respectively). Sloth bears were detected at 76 out of 143 (53.10%) camera trap locations (sites), and independent detections of sloth bear varied from 1 to 15 per site (Fig. 2). However, in the larger grids (12.40 km^2^), the presence of sloth bears was recorded at 51 out of 73 (70.0%) grids. In SNP, a higher number (120) of sloth bear detections was observed compared to 71 detections in DWLS.

### Detection process

The density of fruiting trees significantly influenced the detection probability of sloth bears for Poisson, NB and ZIP distributions (Table 2). The estimated detection probability (*p*) of the best detection model (modelled with the density of fruiting trees) was found to be higher in Poisson (0.08 ± 0.01) than in ZIP (0.06 ± 0.02) and NB (0.01 ± 0.004) distributions (Table 2).

**Table 2:** Model parameter estimates (with constant abundance) for the detection probability (*p*) of sloth bear, obtained from the N-mixture model in Sanjay Tiger Reserve, Madhya Pradesh, India, during 2016–2017.

Model description	β estimates	SE (Standard error)	*P* value	AIC	*p*	SE (Standard error)
**Poisson**						
[p(fruit_density)*λ*(.)]	0.27	0.11	0.01 (<0.05)	1,020.22	0.08	0.01
[p(shrub_density)*λ*(.)]	0.16	0.14	0.25 (>0.05)	1,025.15		
[p(termitemounds)*λ*(.)]	0.09	0.11	0.42 (>0.05)	1,025.85		
[p(trapdays_total)*λ*(.)]	0.03	0.14	0.80 (>0.05)	1,026.42		
**NB**						
[p(fruit_density)*λ*(.)]	0.28	0.14	0.04 (<0.05)	991.86	0.01	0.004
[p(shrub_density)*λ*(.)]	0.09	0.17	0.57 (>0.05)	995.44		
[p(termitemounds)*λ*(.)]	0.08	0.15	0.59 (>0.05)	995.47		
[p(trapdays_total)*λ*(.)]	0.07	0.17	0.68 (>0.05)	995.59		
**ZIP**						
[p(fruit_density)*λ*(.)]	0.29	0.11	0.007 (<0.05)	1,017.04	0.06	0.02
[p(shrub_density)*λ*(.)]	0.16	0.13	0.22 (>0.05)	1,022.56		
[p(termitemounds)*λ*(.)]	0.10	0.11	0.377 (>0.05)	1,023.30		
[p(trapdays_total)*λ*(.)]	−0.04	0.16	0.81 (>0.05)	1,024.04		

**Note:**

Three distributions [Poisson, negative binomial (NB) and zero-inflated Poisson (ZIP)] were used to model the detection probability of sloth bear; Covariates considered: fruit_density–density of fruiting trees per 10 m-radius plot around each camera trap location, shrub_density–density of shrubs per 5 m-radius plot around each camera trap location, termitemounds- number of termite mounds per 10 m-radius plot around each camera trap location, trapdays_total–total number of days or occasions for which camera trap (s) was active; AIC, Akaike Information Criterion; Detection probability estimates (*p*) were provided for the best-selected models of each distribution.

### Relative abundance of sloth bears and effects of covariates

**Figure 3:**
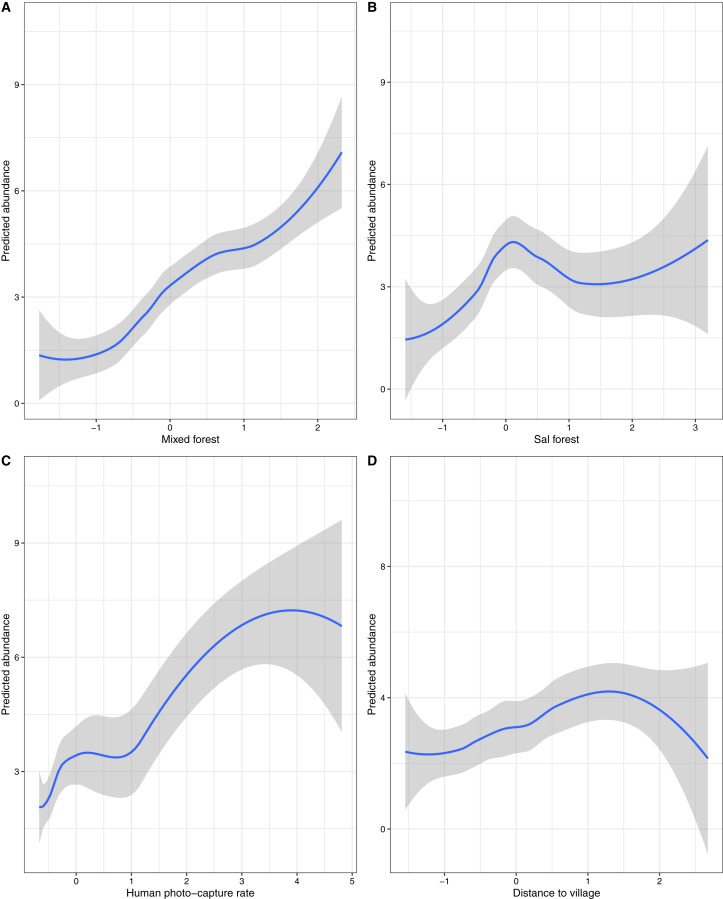
Effects of environmental and anthropogenic covariates on the predicted relative abundance of sloth bears in Sanjay Tiger Reserve, Madhya Pradesh, India, 2016–2017. Covariates considered: (A) Mixed forest, (B) sal forest, (C) human photo-capture rate and (D) distance to village. Prediction of relative abundance was based on the most parsimonious models of Poisson distribution; values of covariates were shown on a standardized scale.

In top-ranked models, the relative abundance of sloth bears was significantly influenced by forest types (mixed and sal forest; environmental), distance to the nearest village (anthropogenic) and human photo-capture rate (anthropogenic) (Tables 3 and 4). In addition to these four, the most parsimonious models also included the LULC types (agricultural land, scrubland and water body); however, none of these covariates significantly influenced the relative abundance of sloth bears (Table 4). Models with NB distributions were the most parsimonious in terms of lower overall AIC values, followed by ZIP and Poisson models (Table 3). Due to uninformative parameters, we did not carry out model averaging for any top-ranked models. Areas of mixed and sal forest predicted a higher relative abundance of sloth bears (Table 4 and Fig. 3; also see Figs. S2 and S3). The distance to the nearest village and human photo-capture rate also had significant positive effects (Table 4 and Fig. 3; also see Figs. S2 and S3) on sloth bear abundance.

**Table 3:** Details of the model selection results of the N-mixture model for estimation of the relative abundance of sloth bears in Sanjay Tiger Reserve, Madhya Pradesh, India, during 2016–2017.

Model description	nPars	AIC	ΔAIC	AICwt	Cumltv-wt
**Poisson**					
(p(fruit_density)*λ*(sal.forest+vildist+human_CR+mixed))	7	998.08	0.00	0.36	0.36
(p(fruit_density)*λ*(sal.forest+vildist+human_CR+mixed+Agricultural.land))	8	999.52	1.44	0.17	0.53
**Null model** (p(.)*λ*(.))	2	1,024.49	26.41	0.00	1.00
**NB**					
(p(fruit_density)*λ*(sal.forest+vildist+human_CR+mixed))	8	983.00	0.00	0.11	0.11
(p(fruit_density)*λ*(mixed +human_CR))	6	983.15	0.15	0.11	0.22
(p(fruit_density)*λ*(mixed+human_CR+Agricultural. land))	7	983.43	0.43	0.09	0.31
(p(fruit_density)*λ*(mixed+sal.forest+human_CR))	7	983.75	0.75	0.08	0.39
(p(fruit_density)*λ*(mixed+vildist+human_CR))	7	983.81	0.81	0.08	0.47
(p(fruit_density)*λ*(sal.forest+vildist+human_CR+mixed+Agricultural.land))	9	984.26	1.25	0.06	0.53
(p(fruit_density)*λ*(mixed+Scrubland+human_CR))	7	984.60	1.59	0.05	0.58
(p(fruit_density)*λ*(mixed+Agricultural.land))	6	984.71	1.70	0.05	0.63
(p(fruit_density)*λ*(mixed+Water.body+human_CR))	7	984.96	1.96	0.04	0.67
(p(fruit_density)*λ*(mixed+sal.forest))	6	984.99	1.99	0.04	0.71
**Null model** (p(.)*λ*(.))	3	993.76	10.76	0.001	1.00
**ZIP**					
(p(fruit_density)*λ*(sal.forest+vildist+human_CR+mixed))	8	995.09	0.00	0.36	0.36
(p(fruit_density)*λ*(sal.forest+vildist+human_CR+mixed+Agricultural.land))	9	996.11	1.01	0.22	0.58
**Null model** (p(.)*λ*(.))	3	1,022.10	27.00	0.00	1.00

**Note:**

Models include the most parsimonious models with the best-selected covariates and null models for the Poisson, negative binomial (NB) and zero-inflated Poisson (ZIP) distributions; Covariates considered: fruit_density- density of fruiting trees, mixed- area of mixed forest, sal.forest- area of sal forest, human_CR- photographic capture rate of humans, vildist–distance to the nearest village, Agricultural.land–area of agricultural land, Scrubland- area of scrubland and, Water.body–area of water body; Model selection was based on number of parameters (nPars), Akaike Information Criterion (AIC), the difference in AIC between best fit models (ΔAIC ≤ 2), AIC weight (AICwt) and cumulative AIC weight of models (Cumltv-wt); all models (including null models) are represented based on the lowest to the highest value of ΔAIC for each distribution.

**Table 4:** Parameter estimates of N-mixture models to determine the relative abundance of sloth bears in Sanjay Tiger Reserve, Madhya Pradesh, India, during 2016–2017.

Model description	β estimates	SE (Standard error)	*P* value
**Poisson**			
Intercept *λ*(.)	0.95	0.25	0.0001 (<0.005)
*λ*(mixed)	0.33	0.10	0.001 (<0.005)
*λ*(human_CR)	0.27	0.08	0.0005 (<0.005)
*λ*(vildist)	0.25	0.11	0.02 (<0.05)
*λ*(sal.forest)	0.21	0.10	0.04 (<0.05)
*λ*(Agricultural.land)	−0.10	0.13	0.47 (>0.05)
**NB**			
Intercept *λ*(.)	2.38	0.35	1.54e−11 (<0.005)
*λ*(mixed)	0.28	0.13	0.03 (<0.05)
*λ*(sal.forest)	0.25	0.15	0.09 (>0.05)
*λ*(human_CR)	0.24	0.12	0.04 (<0.05)
*λ*(vildist)	0.23	0.14	0.09 (>0.05)
*λ*(Agricultural.land)	−0.21	0.16	0.19 (>0.05)
*λ*(Scrubland)	0.14	0.18	0.45 (>0.05)
*λ*(Water.body)	−0.07	0.17	0.66 (>0.05)
**ZIP**			
Intercept *λ*(.)	1.40	0.38	0.0002 (<0.005)
*λ*(mixed)	0.31	0.11	0.004 (<0.005)
*λ*(sal.forest)	0.31	0.14	0.02 (<0.05)
*λ*(human_CR)	0.27	0.07	0.0002 (<0.005)
*λ*(vildist)	0.26	0.10	0.01 (<0.05)
*λ*(Agricultural.land)	−0.14	0.14	0.33 (>0.05)

**Note:**

Estimates were derived from the Poisson, negative binomial (NB), and zero-inflated Poisson (ZIP) distributions for the mean site abundance (Lambda, *λ*) of sloth bears; Covariates considered: mixed- area of mixed forest, sal.forest–area of sal forest, human_CR–photographic capture rate of humans, vildist–distance to the nearest village, Agricultural.land–area of agricultural land, Scrubland- area of scrubland and, Water.body–area of water body.

**Figure 4:**
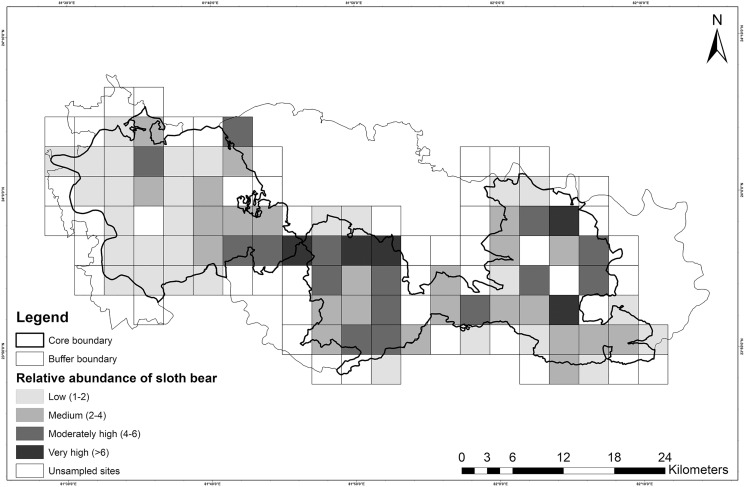
Map showing the relative abundance of sloth bears in Sanjay Tiger Reserve, Madhya Pradesh, India, during 2016–2017; Estimates were based on the most parsimonious Poisson distribution model.

However, the models within ≤2 ΔAIC units for Poisson, ZIP and NB distributions did not pass the GoF test. Overdispersion parameter estimates (ĉ) revealed a marginal lack of fit, which did not appear to differ among Poisson (ĉ = 1.31), NB (ĉ = 1.19) and ZIP (ĉ = 1.28) distributions. Residual diagnostics and mapping of residuals did not show any specific pattern of lack of fit. Hence, this finding could be attributed to unstructured noise or overdispersion rather than a true lack of fit. The NB model (top-ranked) produced unusually high mean site abundance (*λ* = 10.80 ± 3.83) in comparison to mean site abundance estimates of ZIP (*λ* = 4.07 ± 1.56) and Poisson models (*λ* = 2.60 ± 0.64) (Table 5). Spatial variation of relative abundance produced by the Poisson model was less (1–11; see also Fig. 4) than those of the ZIP (1–14) and NB models (4–37).

**Table 5:** Mean site abundance (*λ*) of sloth bears and associated standard error (SE) in Sanjay Tiger Reserve, Madhya Pradesh, India, during 2016–2017.

Model description	Mean site abundance (*λ*) ± Standard error (SE)
**Poisson**	
(p(fruit_density)*λ*(sal.forest+vildist+human_CR+mixed))	2.60 ± 0.64
**NB**	
(p(fruit_density)*λ*(sal.forest+vildist+human_CR+mixed))	10.80 ± 3.83
**ZIP**	
(p(fruit_density)*λ*(sal.forest+vildist+human_CR+mixed))	4.07 ± 1.56

**Note:**

Best-selected models were from three (Poisson, negative binomial (NB) and zero-inflated Poisson (ZIP)) distributions; Covariates considered: fruit_density–density of fruiting trees, mixed–area of mixed forest, sal.forest- area of sal forest, human_CR–photographic capture rate of humans and, vildist- distance to the nearest village.

### Comparing estimates with the RN model

The RN model (null model with Poisson distribution) produced a mean site abundance estimate of 1.62 ± 0.34, which was slightly lower than the estimate produced by the N-mixture model (2.05 ± 0.35). However, the mean site abundance estimate obtained from the N-mixture model was within the 95% confidence interval range (1.08–2.44) of the estimate produced by the RN model.

## Discussion

### Efficacy of the N-mixture model

We reported the first-ever estimates of the relative abundance of the sloth bear using the N-mixture model. We also identified associated factors governing the relative abundance by using count data obtained from the camera trap survey in STR. The N-mixture model can produce reliable abundance estimates if the data meet the assumptions of the N-mixture model while accounting for imperfect detection (Royle, 2004). Also, its ability to model spatial variation of abundance and detection probability as a function of relevant covariates makes its inference more robust and reliable than inferences drawn from conventional index/encounter rate-based surveys (Gilbert et al., 2021). A growing number of studies have assessed the population abundance (relative and absolute) of unmarked individuals by using the N-mixture model from camera trap data (Brodie & Giordano, 2013; Keever et al., 2017; Xiao et al., 2018; Kafley et al., 2019; Penjor et al., 2019) as well as employing other field methods (Belant et al., 2016; Ficetola et al., 2018; Kidwai et al., 2019; Searle et al., 2020). Estimating the abundance of unmarked animals from camera trap data has always been challenging for ecologists. For the past two decades, very few studies have actually assessed the population abundance of Asian bears employing statistically rigorous methods; instead, many of the studies have heavily relied upon sign index or interview-based distributions due to the relatively less expensive nature and ease of application in the field (Garshelis et al., 2022; Proctor et al., 2022). Information on sloth bear abundance and its governing factors is lacking throughout the species’ range (Dharaiya, Bargali & Sharp, 2016). Most of the previous studies (Eisenberg & Lockhart, 1972; Laurie & Seidensticker, 1977; Santiapillai & Santiapillai, 1990; Ratnayeke, Manen & Padmalal, 2007; Akhtar, Bargali & Chauhan, 2008) regarding the abundance of sloth bears produced empirical estimates, except for Garshelis, Joshi & Smith (1999), where the authors first estimated abundance from sound analytical methods in Chitwan. However, in the case of this wide-ranging bear species, site-structured models (*i.e*., the RN model and the N-mixture model) may be reliable for estimating the population trend with careful considerations of model assumptions and study design (Morin et al., 2022).

Moving forward, one should not interpret the abundance estimates obtained from site-structured models as a measure of absolute but relative abundance if the assumption of closure is likely to be violated (Barker et al., 2018; Link et al., 2018; Gilbert et al., 2021). In this context, mean abundance (lambda) from the N-mixture model reflects all individuals using the site (12.40 km^2^ grids in the present study) and not necessarily all individuals restricted to the site. Site abundance estimates obtained from the N-mixture models would be consistently larger than the actual abundance of free-ranging animals like sloth bears, as each site contains multiple overlapping territories of different individual sloth bears. In reality, the actual abundance of animals comes from a considerably larger geographic extent than the actual surveyed area, leading to an overestimation of the mean site abundance and total predicted abundance (Joseph et al., 2009). In the present study, the best-selected model (with NB distribution) in terms of AIC produced substantially larger and ecologically unrealistic abundance estimates of sloth bears compared to ZIP and Poisson models. Mean abundance estimates obtained from NB and ZIP models showed that a large number (~11 and ~4 for NB and ZIP models, respectively) of bears were utilizing each site with considerably low detection probability, which, in general, is unlikely for a species like the sloth bear. In the Indian sub-continent, the lack of studies focusing specifically on the density or abundance of sloth bears and other bear species in a scientifically rigorous framework poses difficulties for comparing our findings to other studies. We expected that for a species like sloth bear, an elusive and solitary ursid, count data obtained from camera traps would be zero-inflated. The NB model performs poorly with a zero-inflated count dataset or when the focal species is not frequently found or is truly absent from a large number of sites surveyed (Joseph et al., 2009). In the present study, NB and ZIP variants of N-mixture models produced lower detection probabilities than Poisson models. Due to low detection probability, N-mixture models can produce positively biased abundance estimates with less precision than other methods (Dénes, Silveira & Beissinger, 2015; Dennis, Morgan & Ridout, 2015). On the other hand, the N-mixture model with a Poisson distribution can handle datasets with excessive true zeros (sites where the species is truly absent), given that the species naturally occurs in low densities (Joseph et al., 2009). In our study, the Poisson model produced a comparatively low mean site abundance (2.60 ± 0.64) by modeling a substantial number (~30% of total sites surveyed) of unoccupied sites. We suggest interpreting the abundance estimates of sloth bears carefully with an ecologically realistic view rather than with strict adherence to the statistical properties of the models.

The mean site abundance of sloth bears produced from the N-mixture model (Poisson distribution) was comparable to estimates obtained from the RN model. Earlier studies have indicated that the RN model would not be a good choice when the focal species is common; in that case, the N-mixture model would be preferable (Dénes, Silveira & Beissinger, 2015; Kéry & Royle, 2015; Gilbert et al., 2021). The RN model reportedly produces imprecise estimates from camera trap-based surveys (covering comparatively small sampled areas), especially if the focal species is cryptic (Gupta et al., 2012; Kalle et al., 2014; Morin et al., 2022). However, there is evidence that the RN model can produce precise abundance estimates, enabling it to detect population trends (O’Brien et al., 2020). Estimates from the RN model can approximate the absolute density of the focal species if the study is conducted on a landscape scale in conjunction with an ecologically relevant grid size for camera trap deployment (Linden et al., 2017). On the other hand, simulation studies have shown that the N-mixture (Poisson and Binomial mixture) model may produce biased estimates of mean abundance in the context of false-positive detections (Nakashima, 2020). However, our findings from both models indicated the overall utility of site-structured models in estimating the relative abundance of elusive species like sloth bears. Although the number of camera trap locations/grid varied (1–4) in our study, the presence of >1 spatial replicates (camera trap locations) within most of the grids (>50%) might have improved the detection and robustness of mean site abundance estimates (Kolowski, Oley & McShea, 2021; Morin et al., 2022).

### Applicability of other methods for unmarked population estimation

Due to the scarcity of spatially explicit studies, we could not directly compare relative abundance estimates of sloth bears with previous findings. Since the primary objective of this study was to understand the applicability of the N-mixture model, we considered other potential methods relevant to the abundance estimation of unmarked animals. However, it has also been found that abundance estimation of unmarked species like Asian bears through camera traps rarely produces precise estimates for conservation planning unless supported by ancillary information obtained from statistically rigorous methods and covering a large number of sampling sites (Morin et al., 2022). Apart from the site-structured models presented here, USCR (*i.e*., the spatial count (SC) model; Chandler & Andrew Royle, 2013) and its extension (*i.e*., spatial presence-absence (SPA) model; Ramsey, Caley & Robley, 2015) can also be applied to estimate the absolute density of cryptic species. The application of the SPA model has been well-demonstrated to estimate the density of small carnivores through camera traps (Chatterjee, Nigam & Habib, 2020a, 2020b). However, these models are computationally intensive (requiring Bayesian analysis) and sensitive to the appropriate choice of priors which could be challenging for a species like the sloth bear due to limited knowledge of its ranging pattern. Distance sampling through camera traps could also be a promising approach to estimating the density of unmarked animals (Howe et al., 2017), especially if the focal species is less abundant (Palencia et al., 2021). However, this method requires the random placement of camera traps which we could not implement, as this study followed the protocol of All India Tiger Estimation (Jhala, Qureshi & Gopal, 2015). Random placement of camera traps might result in substantially low detections of sloth bears, which may not be conducive for further analysis. The N-mixture model would be particularly suitable where finance and logistics are limiting factors. Moreover, if the study intends to model the spatial variation of abundance (relative or absolute), the N-mixture model is a better choice over other models, as long as it meets the objectives and the interpretation can be plausible.

### Effects of environmental and anthropogenic covariates on relative abundance

In the present study, mixed and sal forests positively influenced the relative abundance of sloth bears. In STR, we calculated the density of termite mounds and fruiting trees, utilizing a 10 m-radius plot around each camera trap location. We recorded a high density (35/hectare) of termite mounds in mixed forest, followed by sal mixed and sal forest (27/hectare and 23/hectare, respectively). Similarly, the fruiting tree density was also higher (208/hectare) in the mixed forest in comparison to sal mixed (197/hectare) and sal forest (100/hectare). Due to fewer plots, we could not calculate meaningful density estimates of termite mounds and fruiting trees for the bamboo mixed forest and grasslands. In STR, a high number of fruiting trees and termite mounds in the mixed forest may explain the positive relationship between bear abundance and mixed forest. We found a similar observation from Panna National Park in central India, where the dense forest (comprised of miscellaneous tree species) had the highest densities of fruiting trees and prey insect colonies, with sloth bears preferring this habitat significantly (Yoganand, 2005). However, the limited number of surveyed plots (only at camera trap locations) to support our findings may not be enough to explain the effects of forest types and termite mounds on sloth bear abundance, for which more rigorous sampling is needed. In the North Bilaspur Forest Division, Akhtar, Bargali & Chauhan (2004) found that signs of sloth bears were more common in sal forest than in other forest types. We also found similar results in Chitwan but with distinct seasonal patterns (Garshelis, Joshi & Smith, 1999). The occurrence of bear signs (ground holes and mound holes) in sal forest was frequent in both landscapes, perhaps due to a relatively high number of termite mounds (Garshelis, Joshi & Smith, 1999; Akhtar, Bargali & Chauhan, 2004) and possibly the underground colonies of ants and termites. In the northern part of the Indian sub-continent, termite mounds are more abundant in sal forests (Chakraborty & Singh, 2020). However, underground colonies of termites and ants are difficult to quantify. Hence, we considered the number of termite mounds that are above-ground and visible as a surrogate for the availability of ants and termites (Akhtar, Bargali & Chauhan, 2004; Rather et al., 2020). We also could not find any significant relationship between the relative abundance of sloth bears and local-scale covariates, although the availability of fruiting trees positively influenced the detection probability of sloth bears. In Nepal’s Chitwan and Churia (a lowland forest outside the PAs) forests, the presence of fruiting trees and termite mounds positively influenced the sloth bear’s occupancy and detection probability (Paudel et al., 2022; Pokharel et al., 2022). Das et al. (2014) also recorded a similar observation in southern India during their occupancy-based study. However, we believe that our camera trap-based study design may not be appropriate for drawing inferences on such local-scale covariates, despite their proven importance to the sloth bear’s ecology. The ubiquitous nature of termites and ants in a relatively small area (Redford, 1987) may explain the insignificant relationship between bear abundance and termite mounds. The availability of fruiting trees and termite mounds at a particular point (10 m-radius plot, in our study) may not necessarily represent the entire sampling unit (12.40 km^2^ grids). Thus, it could explain the apparent insignificant contribution of fruiting tree density and termite mounds to the relative abundance of sloth bears.

Sloth bears generally avoid human disturbances (Santhanakrishnan et al., 2015; Puri et al., 2015; Paudel et al., 2022; Pokharel et al., 2022). However, they can also tolerate certain degree of human pressure or get habituated to living in disturbed and fragmented forests (Akhtar, Bargali & Chauhan, 2004; Prajapati, Koli & Sundar, 2021). In the present study, we found that the photographic capture rate of humans and distance to the nearest village positively correlated with relative abundance of sloth bears. However, based on the current study design, it is not known whether a causal relationship between these variables (relative abundance of sloth bear and human photographic capture rate) exists. Moreover, to our knowledge, no other studies have shown that any such ecological relationship prevails. We believe this finding may be purely attributed to the placement of camera traps on the forest roads and trails. Apart from sloth bears (and other carnivores), forest roads and trails are intensively used daily by local people living inside STR. We were interested in this relationship between human occurrence and sloth bear abundance as a matter of concern regarding the possible negative interactions between humans and sloth bears. However, to cope with extensive human pressure in STR, sloth bears have shown a fine-scale seasonal spatio-temporal segregation (Chaudhuri et al., 2022). On the other hand, sloth bears avoided areas close to villages in STR, which is in agreement with previous studies (Santhanakrishnan et al., 2015; Puri et al., 2015; Paudel et al., 2022; Pokharel et al., 2022). Such behavior could be due to the better availability of forage and shelter (day-resting den sites) in relatively undisturbed habitats.

Nevertheless, avoidance of human settlements does not necessarily curb the possibilities of conflict between humans and sloth bears, given the prevailing extensive anthropogenic pressure in STR. Sloth bears are also known to opportunistically raid crops and forage on fruits of *Ziziphus mauritiana, Mangifera indica* and *Syzygium cumini* found in the villages but less abundant inside the forest (Bargali, Akhtar & Chauhan, 2004; Palei, Debata & Sahu, 2020). Habituation to human settlements often leads to severe conflicts between humans and sloth bears (Bargali, Akhtar & Chauhan, 2005; Dhamorikar et al., 2017; Prajapati, Koli & Sundar, 2021), so there are significant conservation implications for this species in human-dominated landscapes.

### Caveats and limitations

Our study has a few limitations and caveats. Firstly, the camera trap design was more oriented towards tiger movement, but also aimed at other co-predators. Secondly, we could not deploy camera traps intensively due to villages and agricultural fields, which resulted in an unequal number of camera traps (1–4) in each of the larger grids (12.40 km^2^). Such design may have affected the detection of sloth bears as they are presumed to raid crops and forage on fruits in the villages situated inside the core area of STR. Thirdly, we restricted the camera trap survey to the core area of STR and therefore, could not account for overall estimates of sloth bears’ relative abundance for the entire STR. Finally, we could not assess the seasonal variation of mean site abundance of sloth bears, as studies conducted elsewhere reported a distinct seasonal variation in sloth bear density (Garshelis, Joshi & Smith, 1999).

## Conclusions

We demonstrated the application of the N-mixture model to estimate the relative abundance of sloth bears from camera trap data. Also, choosing an appropriate probability distribution is crucial to providing ecologically realistic estimates. The N-mixture and RN models with Poisson distribution could be suitable options for estimating the relative abundance of solitary, elusive carnivores, despite the better statistical properties of models with ZIP and NB distributions. However, limited knowledge of ranging patterns of sloth bears in human-dominated landscapes and substantial variation in home range estimates throughout the species’ distributional range could affect the choice of grid size (12.40 km^2^ grids in the current study) for the N-mixture model. If the goal is to detect the population changes over time, site-structured models are rarely effective, especially if knowledge of certain aspects of the species’ ecology (*e.g*., home range, population density) is limited (Morin et al., 2022). Future studies to monitor population changes of sloth bears should focus on the placement (*i.e*., spacing) of camera traps based on a more thorough understanding of their ranging patterns (Fuller et al., 2022). Also, a spatially extensive sampling area with several spatial replicates at each sampling unit (depending on the feasibility and size of the sampling unit) is required to assess the abundance with precision in the case of a low-density population of sloth bears. We highly recommend carrying out density estimation by applying a spatially explicit capture-recapture framework through genetics and comparing results with site-structured models (N-mixture and RN models) and other suitable camera trap-based methods (USCR, SPA, and distance sampling). However, we believe that, in the absence of absolute abundance estimates for an unmarked, habitat generalist, and conflict-prone species like the sloth bear, knowledge of an ecologically realistic relative abundance and associated factors governing the same are imperative for its long-term conservation in a human-dominated landscape.

## Supplemental Information

10.7717/peerj.13649/supp-1 Supplemental Information 1 Forest types of Sanjay Tiger Reserve, Madhya Pradesh, India.

10.7717/peerj.13649/supp-2 Supplemental Information 2 Effects of environmental and anthropogenic covariates on the predicted relative abundance of sloth bears in Sanjay Tiger Reserve, Madhya Pradesh, India, 2016-2017. Covariates considered: A) Mixed forest, (B) Sal forest, (C) Human photo-capture rate and (D) Distance to village. Prediction of relative abundance was based on the most parsimonious models of negative binomial (NB) distribution; Values of covariates were shown on a standardized scale.

10.7717/peerj.13649/supp-3 Supplemental Information 3 Effects of environmental and anthropogenic covariates on the predicted relative abundance of sloth bears in Sanjay Tiger Reserve, Madhya Pradesh, India, 2016-2017. Covariates considered: A) Mixed forest, (B) Sal forest, (C) Human photo-capture rate and (D) Distance to village. Prediction of relative abundance was based on the most parsimonious models of zero-inflated Poisson (ZIP) distribution; Values of covariates were shown on a standardized scale.

10.7717/peerj.13649/supp-4 Supplemental Information 4 Photographic detection matrix of sloth bear for the N-mixture models in Sanjay Tiger Reserve, Madhya Pradesh, India, during 2016-2017.

10.7717/peerj.13649/supp-5 Supplemental Information 5 Covariates were used to model the relative abundance of sloth bears in Sanjay Tiger Reserve, Madhya Pradesh, India, during 2016-2017. All covariates were standardized using the Z score.

## Additional Information and Declarations

### Field Study Permissions

The following information was supplied relating to field study approvals (*i.e*., approving body and any reference numbers): All required permissions for our field surveys were provided by the Madhya Pradesh Forest Department, Madhya Pradesh, India (Letter No: I/3129 dated 29/05/2014).

### Data Availability

The following information was supplied regarding data availability: The raw data is available in the Supplemental Files.
