# Supplementary figures and images for: N-mixture model-based estimate of relative abundance of sloth bear (Melursus ursinus) in response to biotic and abiotic factors in a human-dominated landscape of central India
Source: PeerJ. 2022 Dec 6;10:e13649. doi: 10.7717/peerj.13649 (PMC9745790; doi:10.7717/peerj.13649)

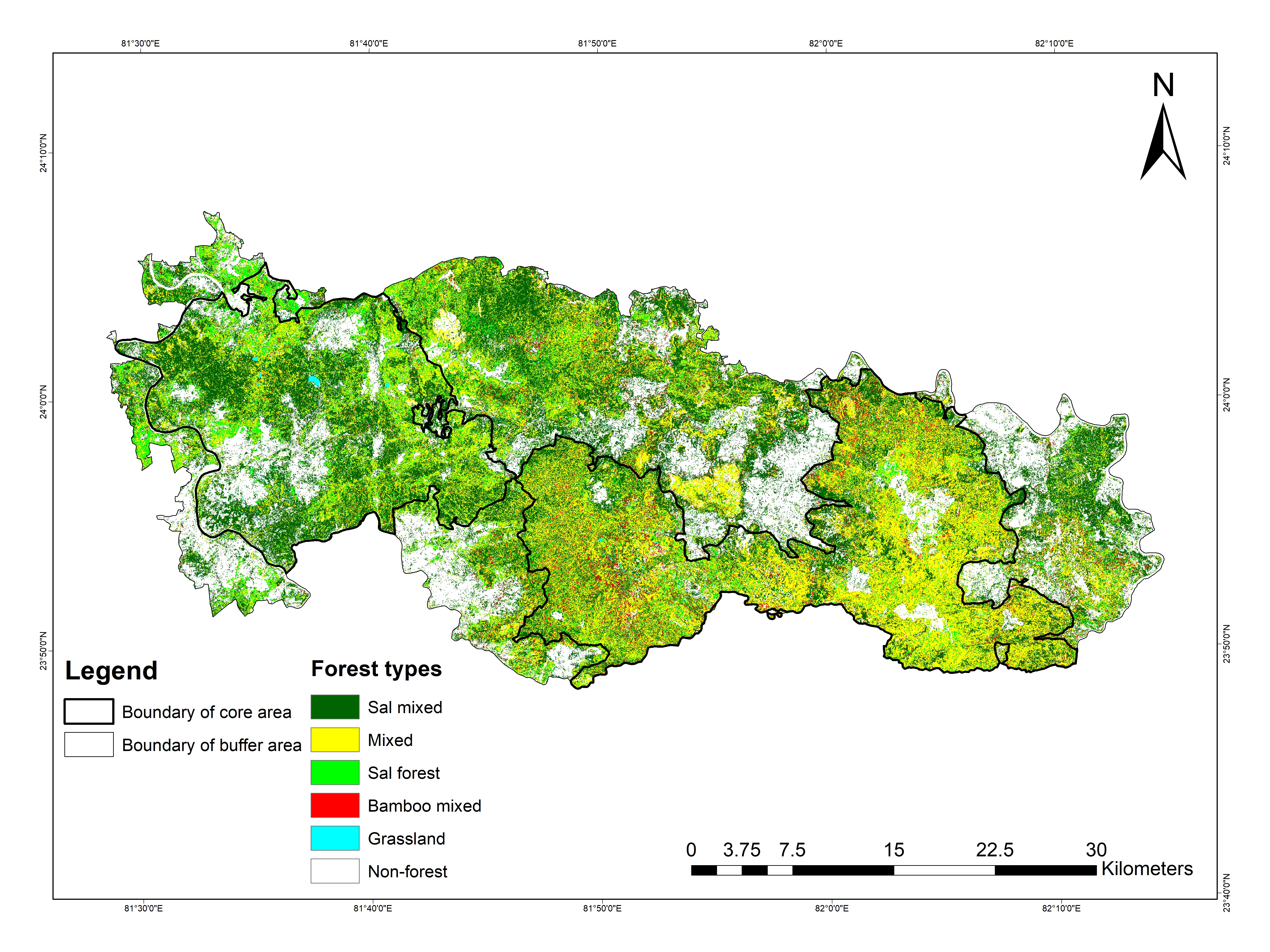

Supplement: Supplemental Information 1 [file peerj-10-13649-s001.png]

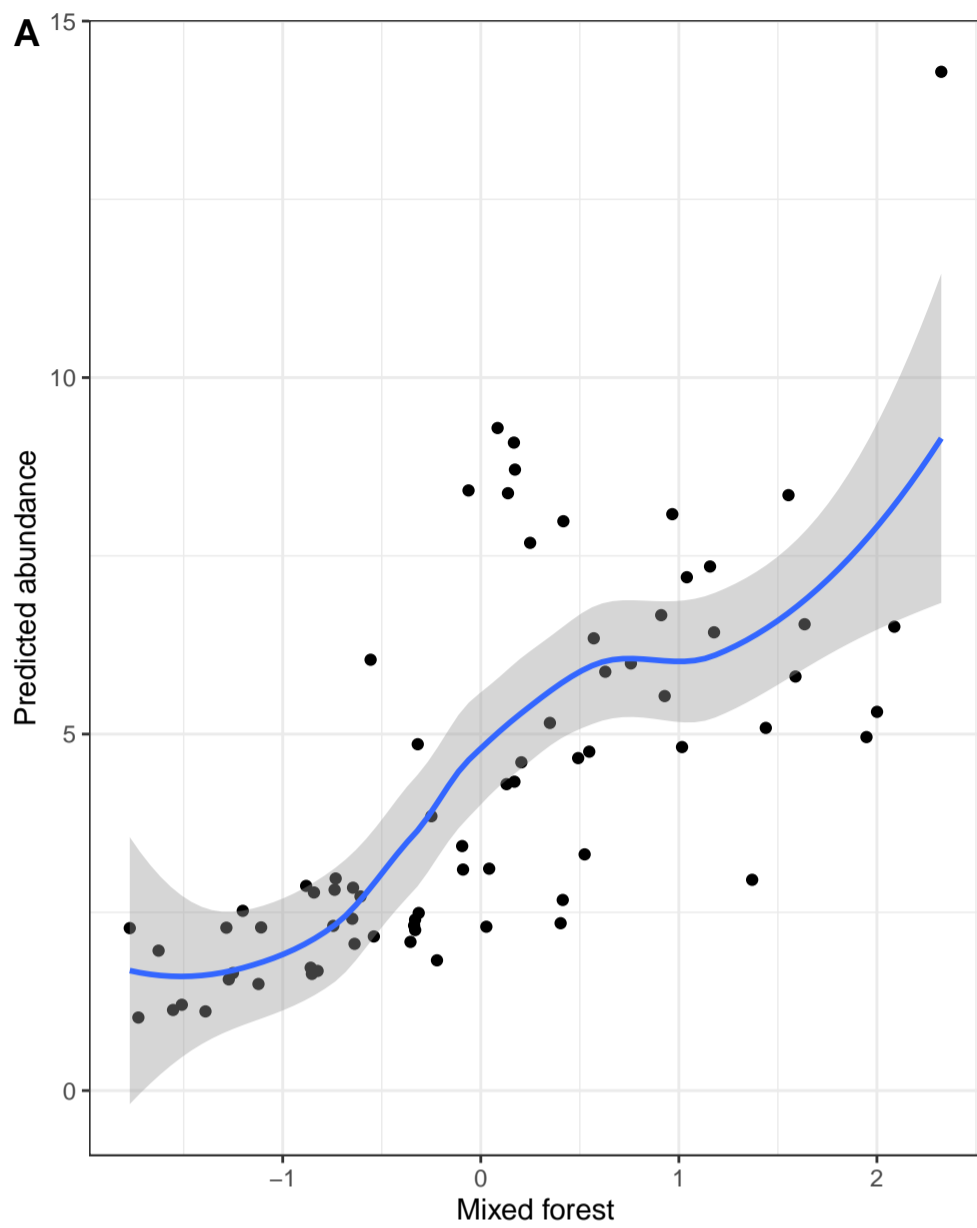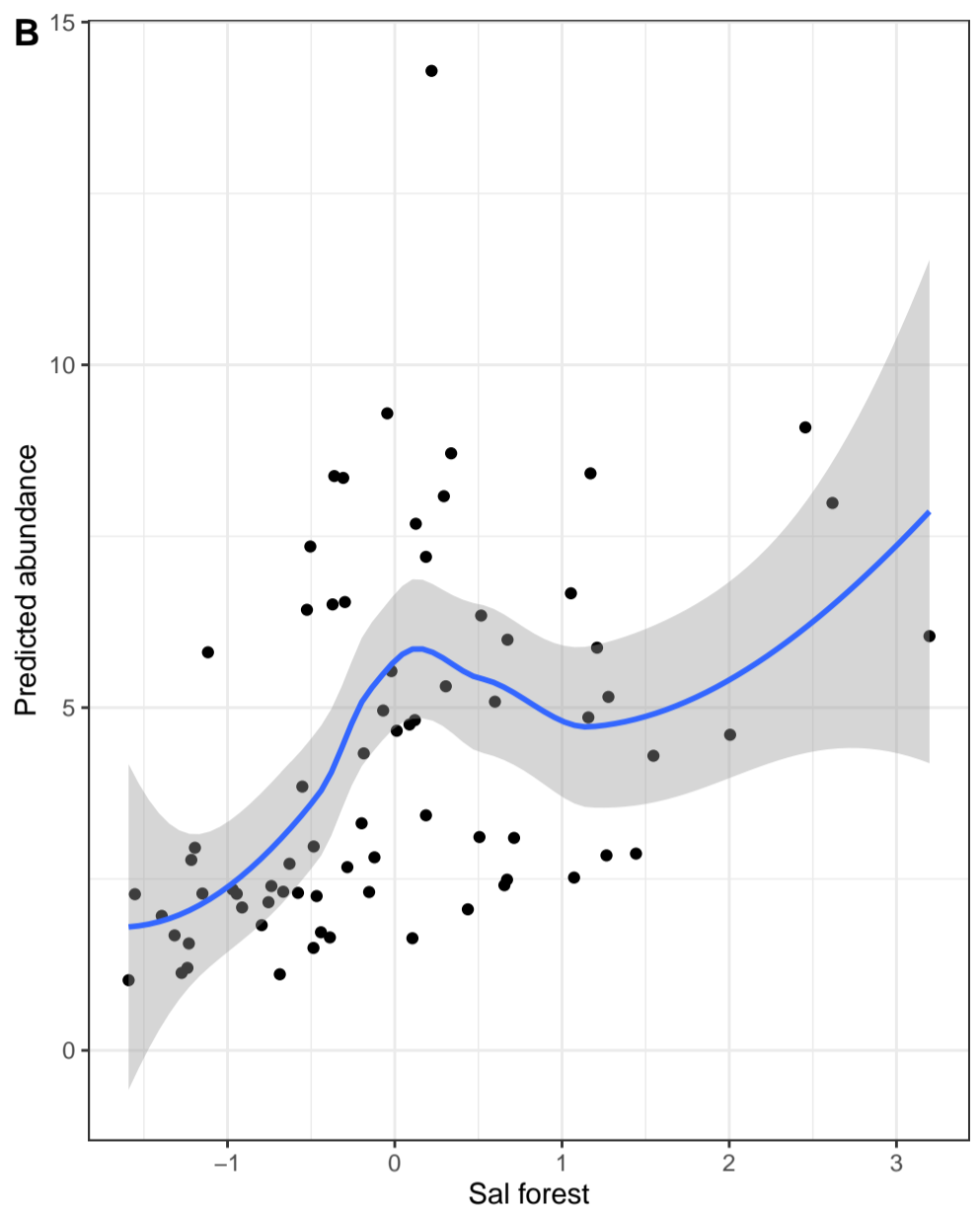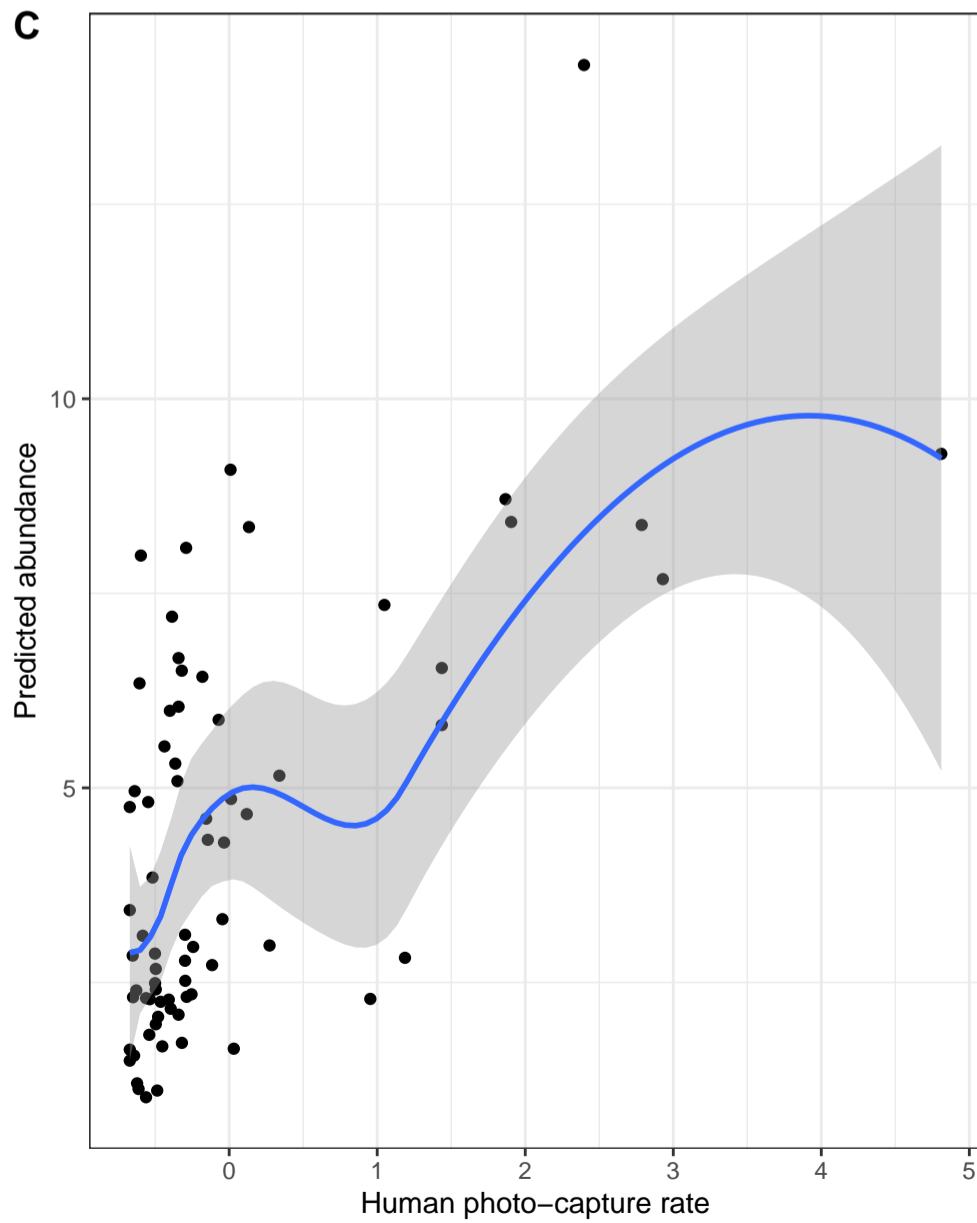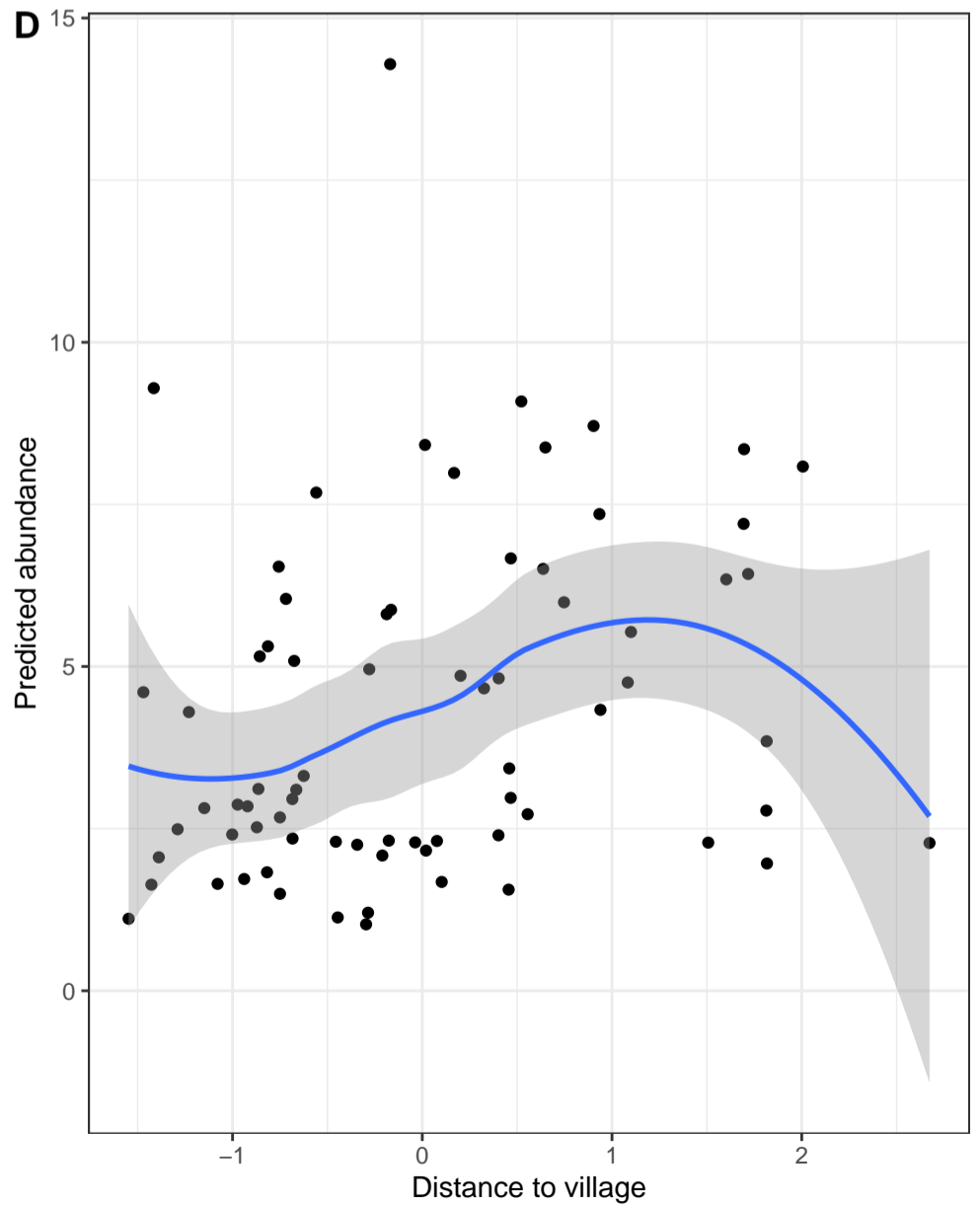

Supplement: Supplemental Information 2 — Covariates considered: A) Mixed forest, (B) Sal forest, (C) Human photo-capture rate and (D) Distance to village. Prediction of relative abundance was based on the most parsimonious models of negative binomial (NB) distribution; Values of covariates were shown on a standardized scale. [file peerj-10-13649-s002.pdf]

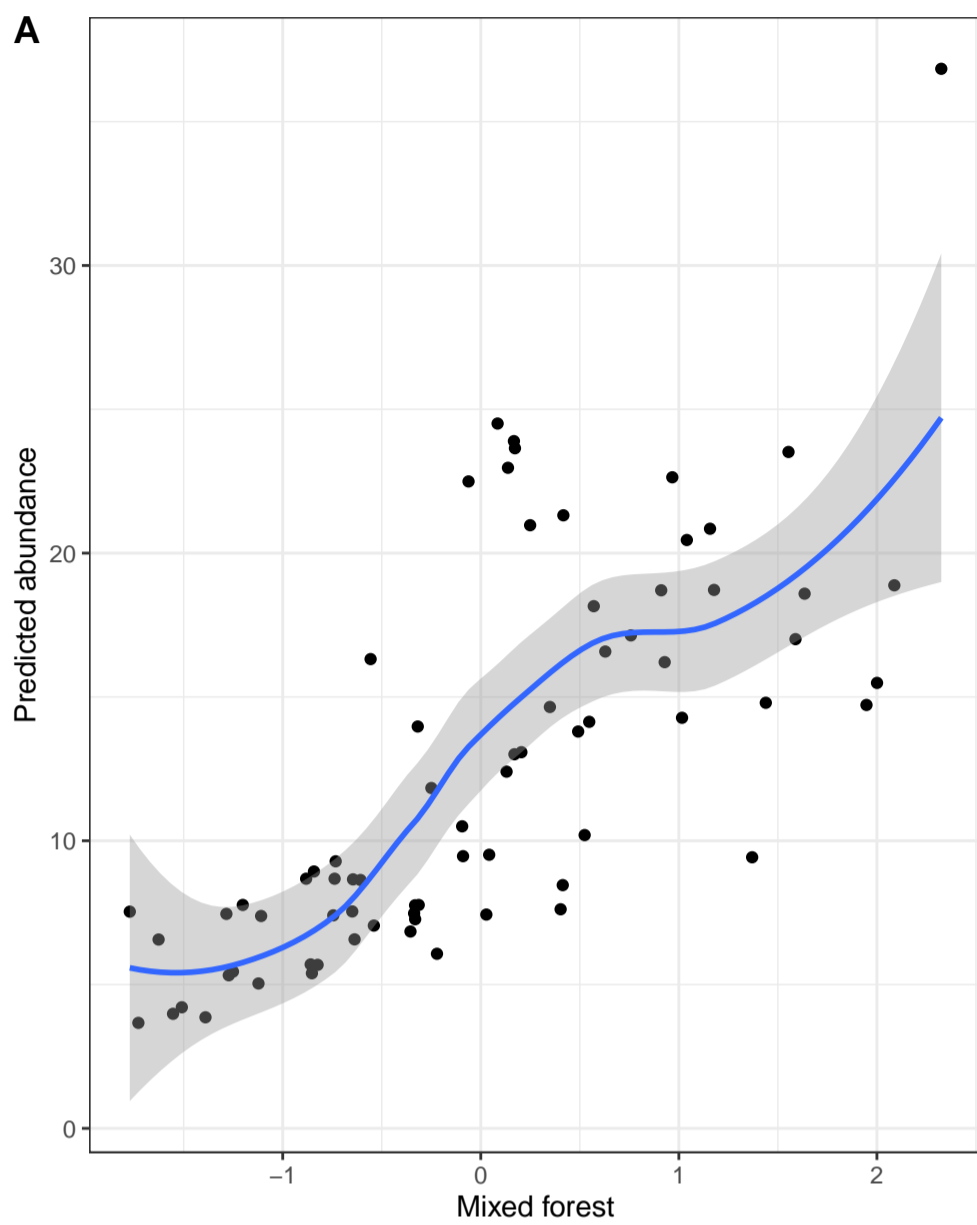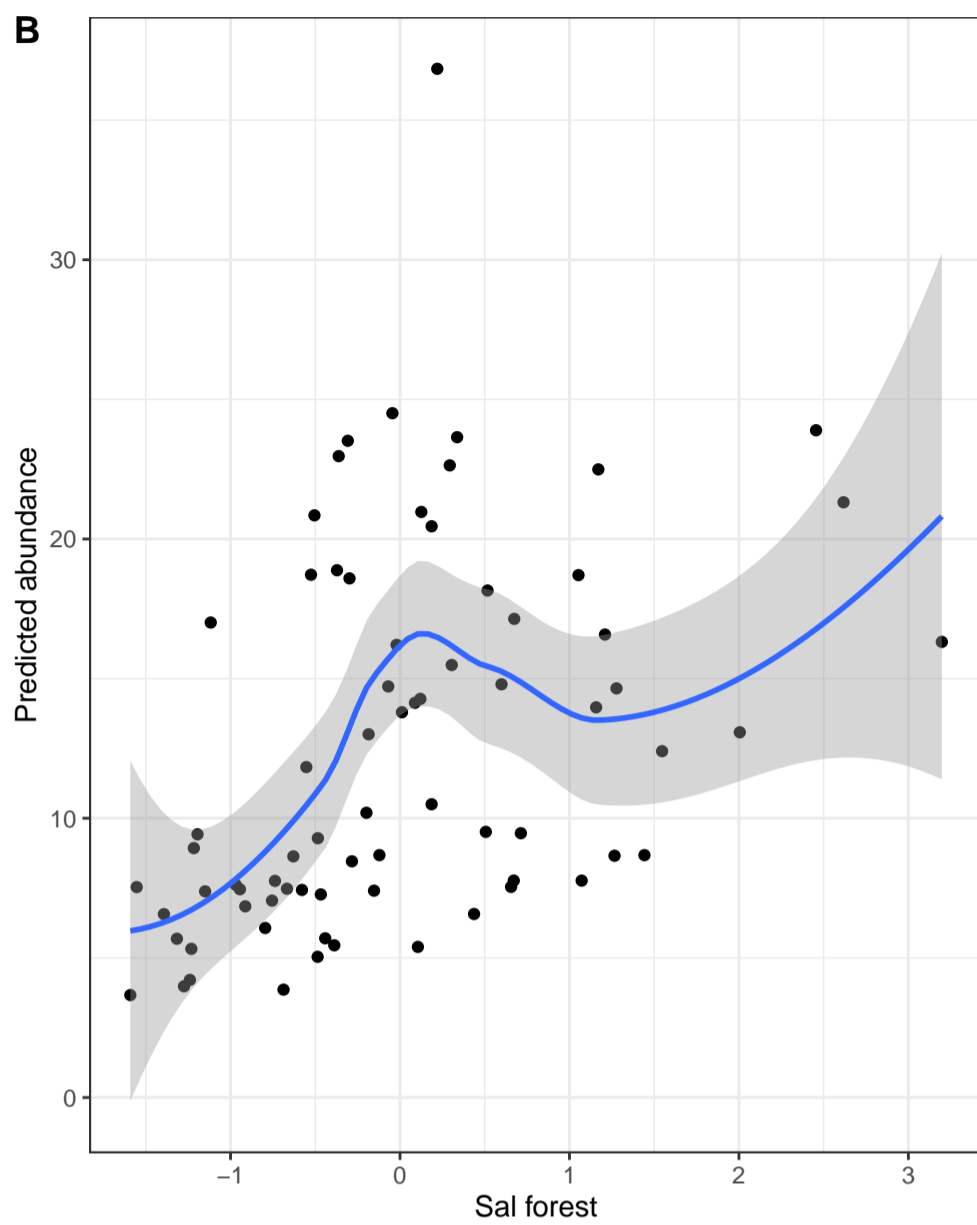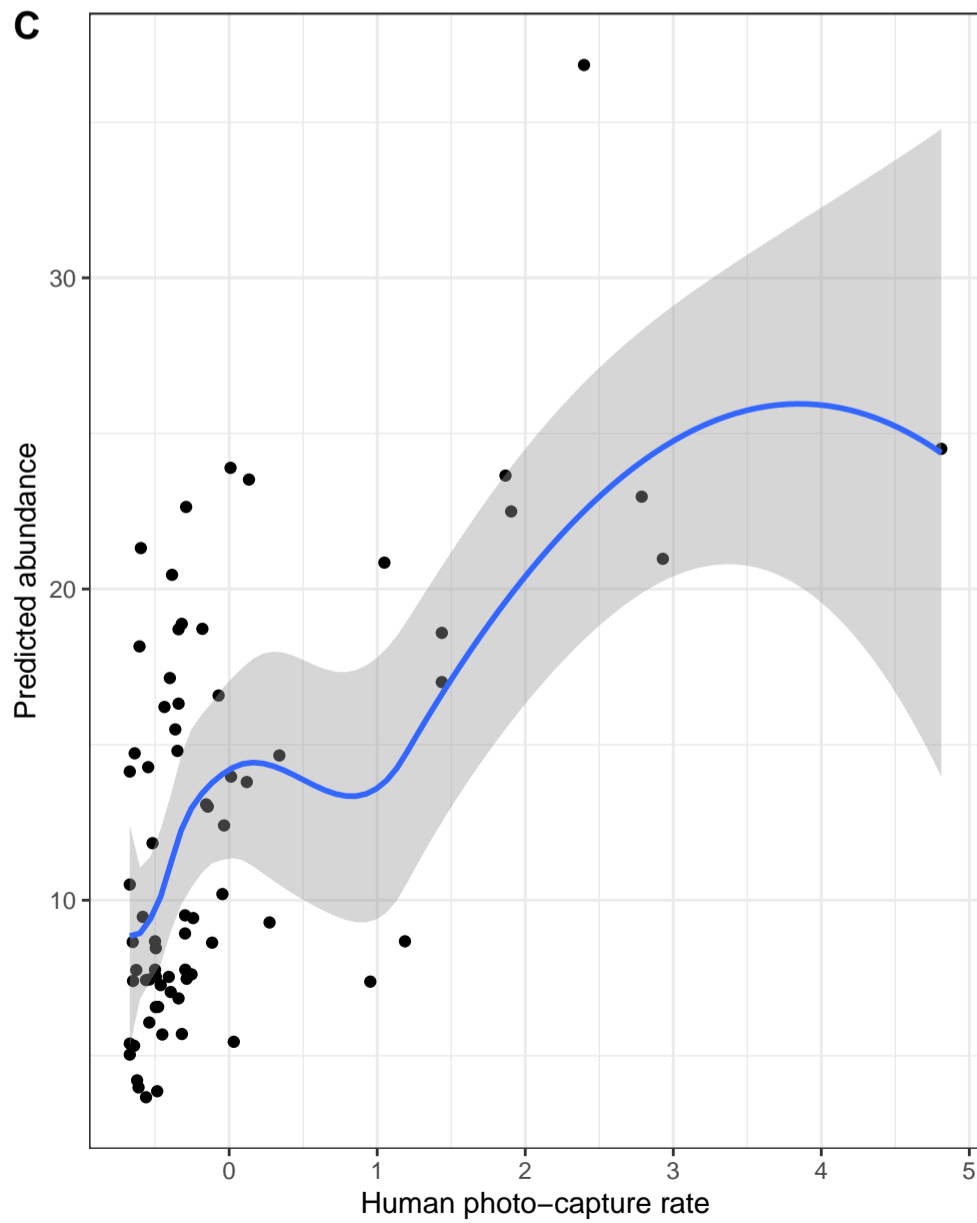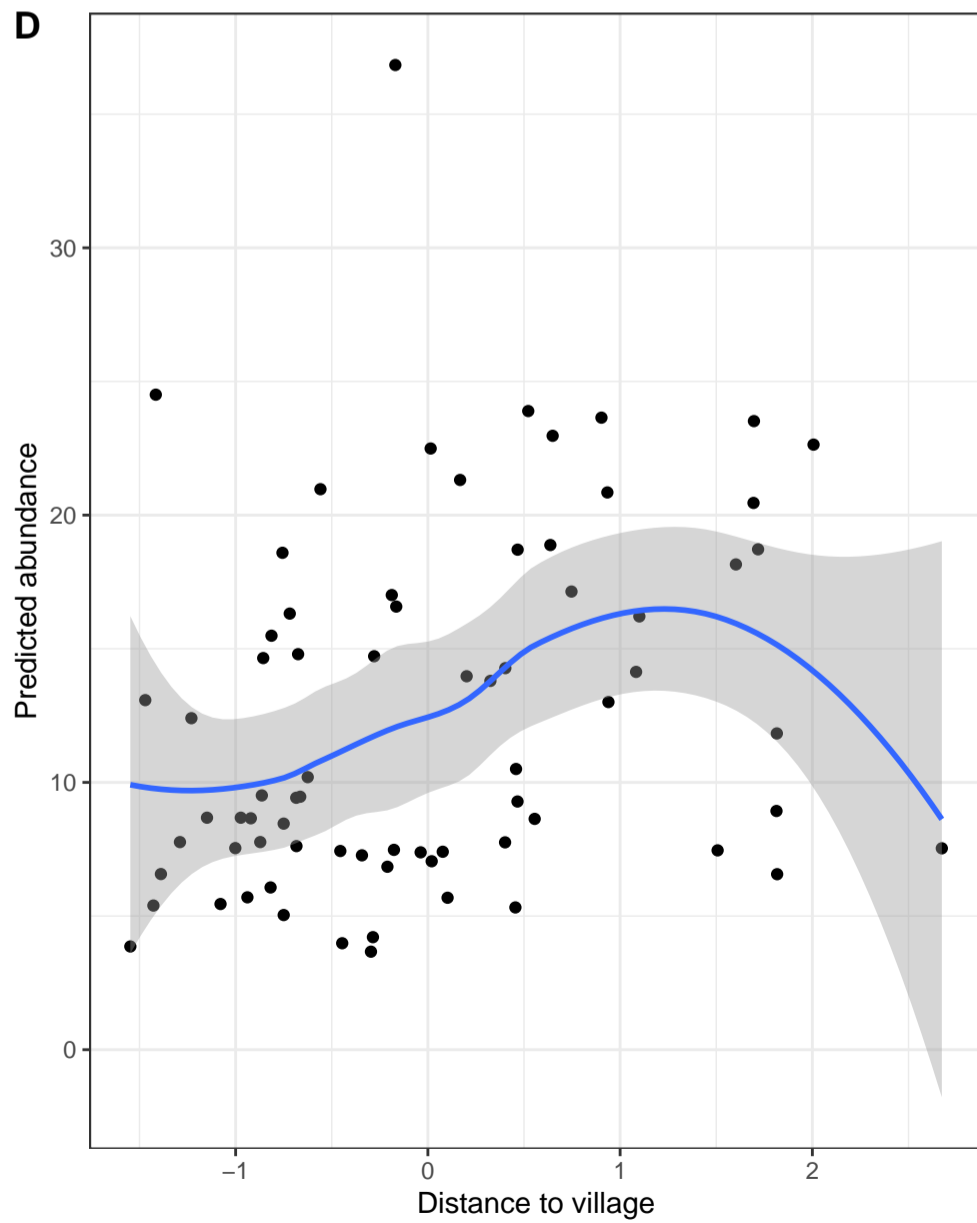

Supplement: Supplemental Information 3 — Covariates considered: A) Mixed forest, (B) Sal forest, (C) Human photo-capture rate and (D) Distance to village. Prediction of relative abundance was based on the most parsimonious models of zero-inflated Poisson (ZIP) distribution; Values of covariates were shown on a standardized scale. [file peerj-10-13649-s003.pdf]
